# Effect of Initial Dissolution Behavior on Cellular Response in Uncoated and Calcium Phosphate-Coated Magnesium Alloy

**DOI:** 10.1021/acsomega.6c04124

**Published:** 2026-09-30

**Authors:** Aoife McFerran, Adam Griebel, Jeremy Schaffer, Ellamay McIlhatton, John O’Connor, Joanna Ward, Jonathan G. Acheson

**Affiliations:** † School of Engineering, 231314 Ulster University, Belfast, BT15 1AP, United Kingdom; ‡ Fort Wayne Metals, Fort Wayne, Indiana 46809, United States

## Abstract

Biodegradable magnesium (Mg) alloys offer a promising alternative to permanent orthopedic implants due to their bone-like mechanical properties, biocompatibility, and complete degradation *in vivo*. However, the rapid dissolution that occurs during the early stages of immersion remains a critical challenge in their feasibility as a resorbable implant. In this work, RF magnetron-sputtered calcium phosphate (CaP) coatings of ∼0.5 μm and ∼1 μm thickness corresponding to 15- and 30-hour deposition times, respectively, were applied to a WE22 alloy. The effect on initial dissolution behavior, ion release, and cellular responses was evaluated when immersed for 1, 7, and 24 h. Uncoated WE22 exhibited rapid dissolution with substantial Mg^2+^ release (∼18 mg/L at 24 h) and raised pH levels from ∼7 to ∼8.8. While ∼0.5 μm CaP coatings reduced these effects, they showed greater mass gain and protein adhesion, suggesting potential coating inhomogeneity or nano-defects trapping corrosion constituents. The ∼1 μm coating provided notable corrosion resistance and maintained a dissolution environment most comparable to physiological conditions. U-2 OS cell viability assessments, including RR assay, DAPI staining, and SEM study, determined that cytotoxicity was concentration-dependent across all time points (*p* < 0.001), with 100% corrosion medium and uncoated WE22 most detrimental to viability. These findings demonstrate the potential for CaP coatings to effectively delay or control the initial corrosion of WE22 while providing enhanced biocompatibility compared to the uncoated substrate.

## Graphical abstract

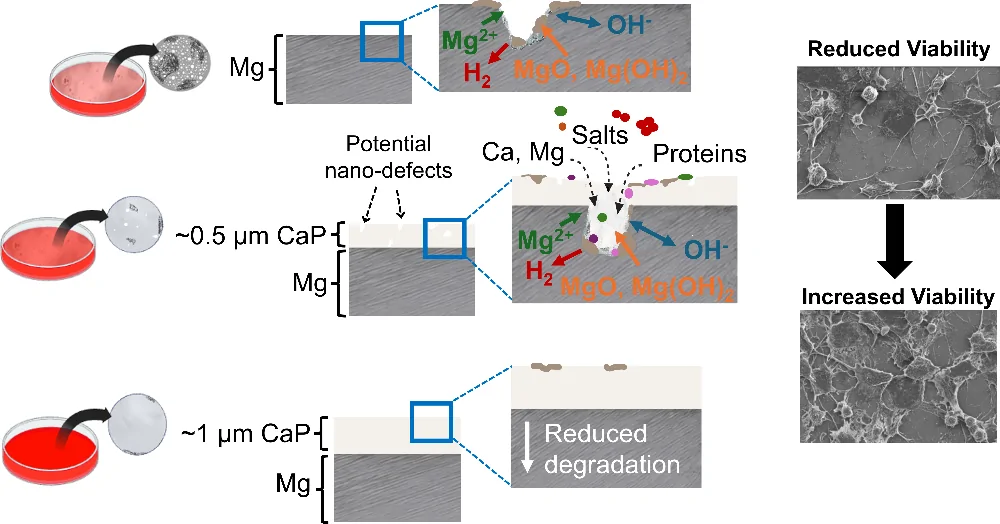

## 1 Introduction

Orthopedic implants have become one of the most rapidly expanding markets over the past two decades,^1^ driven by an increased aging demographic and prevalence of musculoskeletal disorders.^2^ Traditional bioinert and permanent implants, such as titanium alloys, have been favorable in fracture fixation due to their excellent mechanical properties and corrosion resistance;^3,4^ however, there are still several challenges and complications with these materials. The requirement for removal surgeries and subsequent prolonged healing, increased pain, blood loss, and risk of infection,^5^ combined with the additional burden on the healthcare system,^6^ pose significant drawbacks to their use.

These limitations and advancements in biomaterials have led to the development of biomimetic and biodegradable materials. Magnesium (Mg) is a promising biomaterial for temporary orthopedic implants due to its unique properties, its density and elastic modulus are close to that of natural bone, reducing stress shielding, while its biocompatibility and biodegradability allow gradual *in vivo* degradation and replacement by newly formed bone.^7,8^ However, in physiological environments, Mg degrades quite rapidly, resulting in loss of mechanical properties and release of corrosion by-products that may cause cytotoxic effects.^9^ Specifically, rapid corrosion of Mg-based implants is a concern in medical applications because it can lead to excessive release of Mg ions (Mg^2+^), local pH elevation, and hydrogen gas accumulation,^10,11^ all of which may adversely affect surrounding tissue. Although Mg is an essential trace element in the body and plays an integral role in biological functions, such as bone and mineral homeostasis,^12,13^ the effects of rapid Mg^2+^ release can damage surrounding cells and interfere with biochemical processes required for tissue repair.^14,15^

To address these limiting factors, surface modification techniques have been widely utilized, with the application of calcium phosphate (CaP) coatings showing promise in delaying initial corrosion significantly.^16^ Therefore, controlling ion release and reducing reactivity to enhance cellular response, particularly during the early stages of Mg degradation. By not only acting as a protective barrier to the underlying substrate but also mimicking the mineral phase of bone, recent studies have shown CaP coatings to enhance biocompatibility and promote osteointegration between the implant and surrounding tissue.^17^ Improved corrosion resistance combined with enhanced bone integration emphasizes the potential of CaP coatings to extend implant longevity while facilitating effective bone healing and regeneration. Despite these advances, literature remains somewhat variable in alloy composition, coating architecture, and corrosion solution—making direct comparison challenging.

Current studies in the literature mostly emphasisze later-stage degradation or specific *in-vivo* end points, and early dissolution is often unexplored. The early stage of dissolution is specifically important as initial ion release, pH change, and various surface interactions can strongly influence cell behavior and response. *In-vitro* assessment of short-term degradation therefore provides a useful and controlled approach to review the initial material-environment interactions that occur before the development of more complex biological responses. Moreover, the use of fully supplemented McCoy’s 5A medium as a degradation solution provides a more biologically relevant environment due to the presence of proteins, amino acids, and other organic components.

The work presented provides an overview of the early degradation behavior of an uncoated WE22 Mg alloy and CaP-coated WE22 over immersion periods of 1, 7, and 24 h. Additionally, the associated cellular response to dissolution processes and environmental conditions was investigated to relate degradation behavior to biocompatibility and cytotoxicity. Few studies have focused on the first 24 h of degradation in WE22 Mg alloys with concurrent biological assessment. Therefore, this study addresses an important timeframe of degradation and its influence on biological response, while evaluating if CaP coatings with varying thicknesses can improve short-term use of WE22 in a more physiologically relevant environment.

## 2 Methods and Materials

### 2.1 Sample Preparation

A custom medical grade Mg alloy, WE22, with a composition of 96% Mg and 4% rare earth elements (Fort Wayne Metals, USA), was utilized to create coupons of 10 mm diameter and 1 mm thickness. The coupons were abraded on all faces using p1200 SiC paper (RS Components, UK) and ultrasonically cleaned in 99% isopropanol (Sigma-Aldrich, UK) for 10 min before drying and storing at 10% relative humidity.

### 2.2 RF Magnetron Sputtering Process

The RF magnetron sputtering process was conducted with a custom-built high-vacuum physical deposition system and includes two torus mag keeper circular magnetron sputtering sources (Kurt J. Lesker, USA) situated below a rotating sample holder. Sputtering settings, described in Table 1, were utilized to deposit CaP coatings onto all faces of the WE22 alloy coupons.

**Table 1:** List of Sputtering Parameters Used to Deposit CaP Coatings

**Sputtering Parameter**	**Setting**
Power	150 W
Ramp-up power	0.3 u/s
Time	15 and 30 h
Chamber pressure	5 × 10^–5^ Torr
Working gas	Argon (99.995%)
Gas flow rate	32 – 35 sccm
Throw distance	150 mm

### 2.3 Immersion Setup

The dissolution of uncoated and CaP coated WE22 alloys was investigated when immersed in McCoys 5A medium with l-glutamine (Sigma Aldrich, USA). The medium was supplemented with 10% foetal bovine serum and 1% penicillin/streptomycin mix (5000 units: 5 mg/mL, respectively). Prior to experimentation, samples were sterilized in a Class II biosafety cabinet (Bioquell UK Ltd., UK) under UV light for 30 min on each side of the coupon. The volume of test solution was calculated according to the standard ASTM G31-21 ^18^ which informs the solution volume to specimen surface area ratio as 0.2 mL/mm^2^. The medium was warmed to 37.5 °C in a water bath prior to use; it was then transferred to sterile containers, and the WE22 coupons were carefully added. The sterile containers, with medium and substrate, were incubated statically under standard conditions at 37.5 °C and 5% CO_2_ until the desired time point was achieved (1, 7, or 24 h). Upon removal from container, samples were weighed with a microbalance and carefully placed in a 24-well plate and stored at 10% relative humidity.

### 2.4 Characterization

#### 2.4.1 Line Profilometry

Assessment of coating thickness on silicon (Si) wafers was reviewed by stylus profilometry using a Dektak XT system (Bruker, USA). A section of the Si wafer was masked with aluminum foil before sputtering so that the height difference could be analyzed by a diamond conical tip. This system was also used to review surface roughness on the uncoated and CaP-coated WE22 coupons. For each sample, five scans were performed across various areas of the Si wafer and WE22 coupons using the parameters in Table 2.

**Table 2:** List of Parameters Utilized to Assess Coating Thickness on Si Wafers and Coating Roughness on WE22 Coupons

Stylus Parameter	Setting
Range	65.5 μm
Stylus Type	2 μm
Stylus Force	3 mg
Length	5000 μm
Duration	60 sec
Resolution	0.278 μm

#### 2.4.2 Optical Microscopy

Optical microscopy was performed using a SmartZoom 5 microscope (Zeiss, Germany) with a 1.6x objective. Optical micrographs of samples were obtained pre- and postimmersion in medium to evaluate changes in surface morphology and topography caused by degradation processes.

#### 2.4.3 X-ray Diffraction (XRD)

XRD measurements were conducted on uncoated WE22, ∼0.5 μm and ∼1 μm CaP coated WE22 using a 3^rd^-Generation Empyrean III diffractometer (Malvern Panalytical, Netherlands). The instrument was operated at 45 kV and 40 mA with Cu Kα radiation (λ = 1.5406 Å). All XRD measurements were performed using a grazing-incidence configuration (GI-XRD) to enhance surface sensitivity and minimize diffraction contributions from the underlying substrate. A fixed incident angle (ω) of 1.0° was used for all scans. Diffractograms were collected over a 2-theta (θ) range of 20°– 60° using a step size of 0.02°, with a counting time of 4 seconds per step. These parameters were chosen to provide sufficient resolution and signal-to-noise for thin-film CaP crystallinity assessment.

#### 2.4.4 Scanning Electron Microscopy/Energy Dispersive X-ray (SEM/EDX)

A Hitachi SU5000 scanning electron microscope (SEM) was used to examine the surface topography of uncoated and coated substrates. Micrographs were obtained under high vacuum and in secondary electron (SE) mode, with accelerating voltages ranging from 5 kV to 10 kV to maximize surface sensitivity and a spot size of 10 nm. Energy-dispersive X-ray (EDX) analysis was also conducted through Aztec software (Oxford Instruments, UK) using an accelerating voltage of 5 kV to generate a beam spot size of 50 nm, and three areas of each surface were assessed at a resolution of 1024 × 1024. The elemental maps were then used to obtain the atomic percentage (at %) composition and calculate the Ca/P ratio of coating.

#### 2.4.5 Fourier-Transform Infrared – Attenuated Total Reflectance (FTIR-ATR)

FTIR-ATR was implemented to identify chemical bonds associated with proteins in McCoy’s 5A medium and phosphate from CaP coated samples. The resulting spectrum produces a unique fingerprint profile to identify such bonds. This was completed by a Varian 640-IR FT-IR Spectrometer (Varian, USA) with a diamond ATR accessory. Absorbance was measured between wavelengths of 4000-400 cm^–1^ at a resolution of 2 cm^–1^ and a total of 50 scans per sample.

#### 2.4.6 Atomic Absorption Spectroscopy (AAS)

A Perkin Elmer AAS instrument (Perkin Elmer, UK) was used with a hollow cathode lamp for Mg, utilizing a wavelength of 285.21 nm and current of 6 mA. Acetylene was used as the burner gas at a flow of 2.5 L/min and a slit width of 0.7 nm to ensure spectral resolution. Reagent blank (McCoy’s 5A medium) and samples (corrosion medium) were diluted to 1 in 40 mL with DI water with a working volume of 10 mL. Following this, 1 mL of a 1% lanthanum chloride solution was added to reagent blank and samples to reduce chemical interference. A calibration curve was generated using Mg stock solutions of 0.5, 1, and 2 parts per million (PPM).

### 2.5 Cell Culture

#### 2.5.1 Cell Count and Seeding

Cell studies were conducted using a McCoys 5A medium, as described previously. An immortalized human osteosarcoma cell line (U-2 OS) was selected as a representative model for human bone cells. Cells were routinely monitored under an inverted microscope (Nikon, UK) and sub-cultured every 4-5 days to maintain a confluency of ∼70%. All cell culture and biological assays were performed aseptically in a biosafety cabinet.

The test immersion solution for 1, 7, and 24 h was diluted, if necessary, to achieve working concentrations of 100, 50, 25 and 1%. Each of the solutions was stored under standard conditions until required. Prior to performing *in-vitro* studies, glass coverslips were wiped with 70% IMS and treated under UV light for 10 min either side for sterilization. They were then transferred to 24-well tissue culture plastic plates and stored in a biosafety cabinet. The cell suspension concentration was determined using an automated Scepter 3.0 cell counter (Sigma-Aldrich, UK). The suspension was standardized to 3 × 10^5^ cells per mL (15,000 cells per substrate); 50 μL of this cell suspension was then pipetted onto the center of each glass coverslip and incubated for 2 h under standard conditions. A further 950 μL of standard medium was added into each of the wells of negative and positive controls. The adjusted medium from each sample type was then added to the sample wells. All plates were incubated until each of the desired time points (days 1, 3, and 7).

#### 2.5.2 Resazurin Reduction Assay to Measure Cell Viability

Cell viability at days 1, 3, and 7 was assessed using a resazurin reduction (RR) assay, a method for quantifying viable cells and based on the reduction of non-fluorescent blue resazurin to fluorescent pink resorufin by the mitochondria of metabolically active cells. Resazurin salt (ab145513-5g. purity >80%, Abcam, UK) was dissolved in 0.01 M phosphate-buffered saline (PBS) to obtain a concentration of 0.30 mg/mL, then sterile filtered through a 0.2 μm membrane into a light protected container. Samples with their culture media were placed in 24-well plates, and 50 μL of the RR solution was added to each well. Plates were wrapped in aluminum foil, rotated at 20 rpm for 5 min at room temperature, and incubated for 4 h. Wells were thoroughly mixed after incubation and three separate 100 μL aliquots per well were transferred to black 96-well plates, which were protected from light until measurement was completed. Fluorescence was recorded using a Tecan Spark multi-mode plate reader (Tecan Group Ltd., Switzerland) using 560 nm excitation/590 nm emission filter set.

#### 2.5.3 Scanning Electron Microscopy of U-2 OS Cells

All culture medium was aspirated from control and test substrates, and each well was washed twice with 0.01 M PBS followed by three washes with deionized water to minimize PBS crystallization. Cells were then fixed by adding 2.5% glutaraldehyde (Sigma-Aldrich, UK) for 45 min at room temperature, once glutaraldehyde had fixed the cells, it was then aspirated. Gradual dehydration of samples was achieved by using an alcohol series; this was completed by sequentially adding concentrations of 25, 50, 75, 90, and 100% for 8 min each at room temperature, with aspiration between each of the concentrations. After ethanol dehydration, a 1:1 ratio of ethanol and hexamethyldisilazane (HMDS) (Sigma-Aldrich, UK) was added for 8 min at room temperature, and then 2-3 drops of HMDS were added to thoroughly dry samples overnight. Each fixed glass coverslip was then coated with a conductive layer of gold/palladium using a sputtering system (Emitech K500X, Quorum Technologies, UK) at 25 mA for 2 min before reviewing via SEM.

#### 2.5.4 4′,6-Diamidino-2-Phenylindole (DAPI) Staining

Culture medium was aspirated from each well, and substrates were carefully rinsed twice with 0.01 M PBS. Following this, 4% paraformaldehyde (PFA) in 0.01 M PBS was added for 8 min at room temperature and then washed twice with 0.01 M PBS before staining with 300 N m 4′,6-diamidino-2-phenylindole (DAPI) in 0.01 M PBS for 10 min at room temperature. Prior to imaging, samples were washed a further three times with 0.01 M PBS. A wide-field fluorescent microscope and associated software (Zeiss Axio Imager and Zen software, Zeiss, Germany) was used to image the fluorescently stained ‘blue’ nuclei with a 10x objective. At least three random areas per DAPI-stained sample were imaged using Colibri 7 LED illumination at an excitation wavelength of 405 nm. DAPI-stained micrographs were analyzed to quantify monolayer coverage (%) for each sample. Images were converted to 8-bit grayscale and thresholded to distinguish between cellular nuclei and background, followed by calculating the stained nuclei coverage.

#### 2.5.5 Statistical Analysis

For the resazurin reduction assay, the cell viability data was pre-processed by subtracting the negative control from all sample fluorescence values (including from the positive control data). The results were then normalized to the positive control using the formula below:

Aliquots of the same sample were averaged. After preprocessing, the values could be interpreted relative to the positive control (e.g., a value of +5 indicates the viability was 5% higher than the positive control, and a −5 indicates the viability was 5% lower than the positive control). To assess the relationship between the relative viability and experimental variables (sample type, media concentration, and corrosion time), a linear regression model was fitted to the data in R (version 4.5.1).^19^ An interaction term between corrosion time and sample type was added to the model; this was because the relationship between relative viability and corrosion time was dependent on sample type (this can be seen in Figure 11 where the green and blue lines have different slopes). The model was interpreted by the coefficients, *p* values, and R-squared value. Predictions from the models were produced using *emmeans* ^20^ for visual interpretation (using *ggplot2*)^21^ and representative data. Full model results are presented in the Appendix.

All other statistical analyses were conducted using a paired, two-tailed *t*-test with outcomes determined via *p*-values where <0.05, 0.01, and 0.001 are denoted by *, **, and ***, respectively.

## 3 Results

Figure 1 demonstrates the relationship between coating time and mean coating thickness of CaP sputtered coatings on Si wafers and WE22 substrates. At shorter deposition times of 5, 10, 15, and 30 h, coating thickness could only be determined on silicon wafers due to the roughness of WE22 alloy. As coating time increased, a linear progression in coating thickness was observed on Si wafers, increasing from ∼0.17 μm at 5 h to ∼0.93 μm at 30 h at 150 W. At 90 h, thickness could then be measured on the Mg substrate; this was determined as ∼2.3 μm, closely aligning with the Si wafer measurement of ∼2.2 μm at the same deposition time.

**Figure 1:**
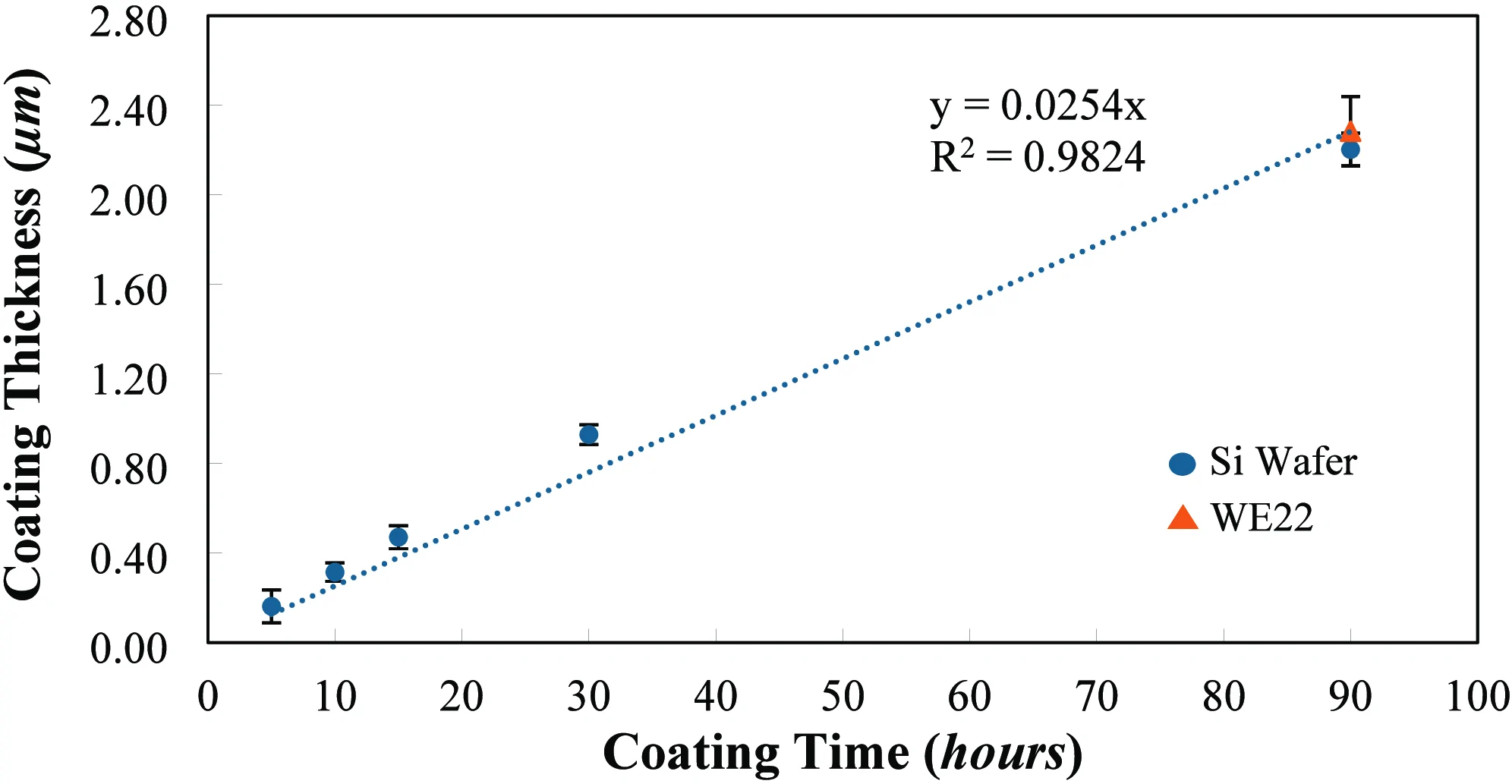
Mean coating thickness of CaP coatings sputtered for 5, 10, 15, 30, and 90 h on Si wafers and 90-hour coating on WE22.

The micrographs in Figure 2 show the topography of the ∼0.5 μm and ∼1 μm CaP coatings on WE22. Low magnification micrographs, Figure 2a and Figure 2c at x1k, show the overall surface topography of the CaP coated substrates, whereas higher magnification micrographs, Figure 2b at x40k and Figure 2d at x25k, reveal distinctive features of the ‘snowballing’ effect that is characteristic of amorphous/semi-crystalline CaP coatings fabricated by magnetron sputtering. This ‘snowballing’ morphology is more prevalent in the ∼1 μm thick coating and forms a denser coverage compared to ∼0.5 μm coating.

**Figure 2:**
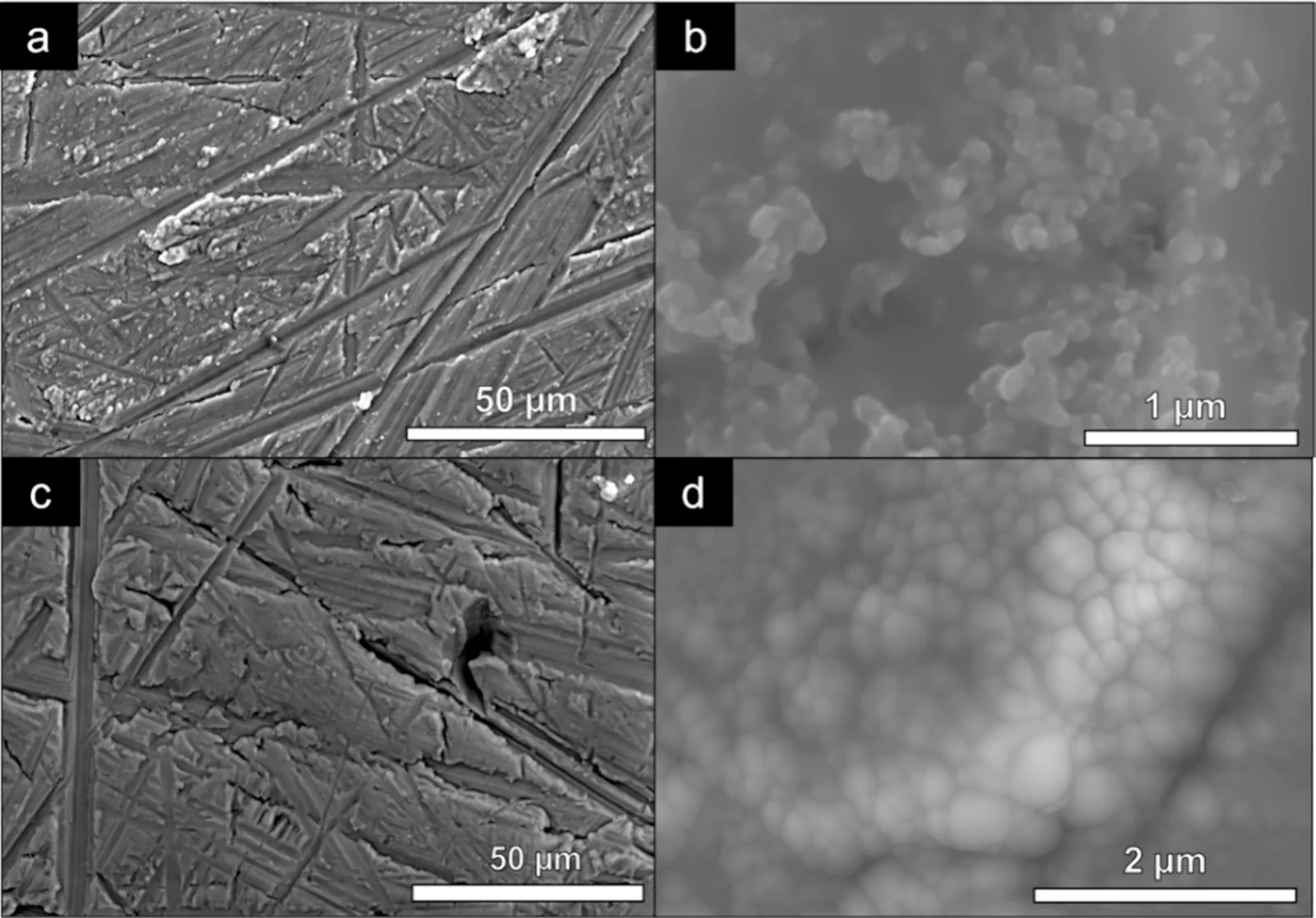
Micrographs of CaP coatings on WE22 with thicknesses of (a,b) ∼0.5 μm and (c,d) ∼1 μm.

From GI-XRD analysis, illustrated in Figure 3, characteristic peaks of the α-Mg phase in the WE22 Mg substrate are observed at 32.2° (100), 34.4° (002), 36.6° (101), 47.8° (102), and 57.4° (110), confirming the presence of the metallic phases (taken from JCPDS card 35-0821). As the coating was deposited, and coating thickness increased, the intensity of the Mg-related peaks progressively decreased, indicating less diffraction due to greater coating thickness. Due to the amorphous nature of the CaP sputtered coating, there are no additional crystalline CaP peaks observed.

**Figure 3:**
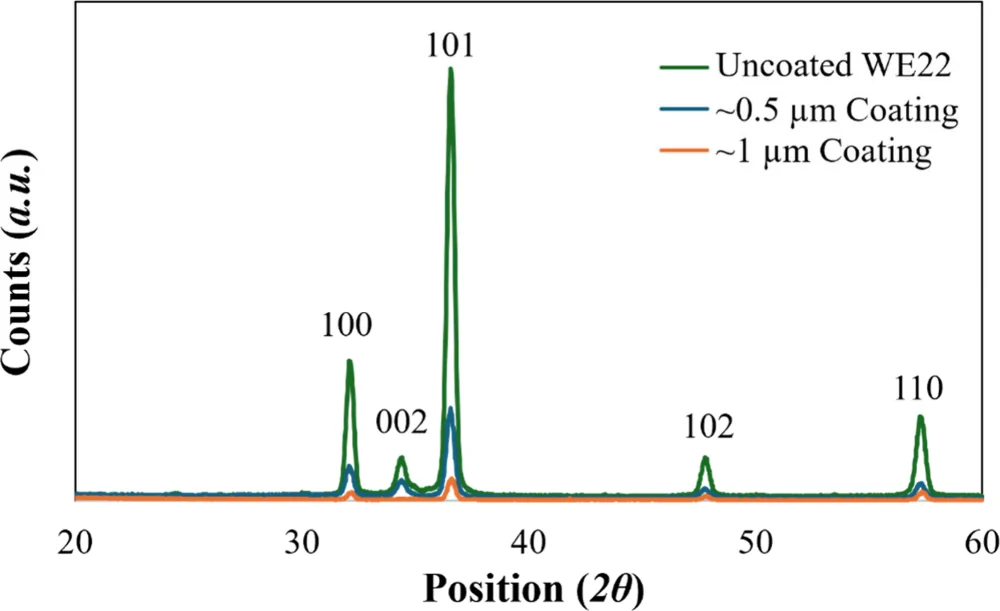
GI-XRD scan for uncoated, ∼0.5 μm and ∼1 μm CaP coated WE22; peaks labelled are α-Mg phases present in WE22 alloy.

The influence of CaP coatings on surface roughness of WE22 was evaluated using Pa (mean height) and Pq (root mean square roughness). Surface roughness measurements of uncoated WE22, ∼0.5 μm and ∼1 μm CaP coated WE22 are displayed in Figure 4. There are no significant differences between uncoated and ∼0.5 μm CaP coated samples, which indicates that shorter deposition times do not substantially change the topography of the substate. In contrast, the ∼1 μm CaP coated samples did reduce surface roughness, producing a smoother topography of ∼0.22 μm and ∼0.28 μm for Pa and Pq, respectively.

**Figure 4:**
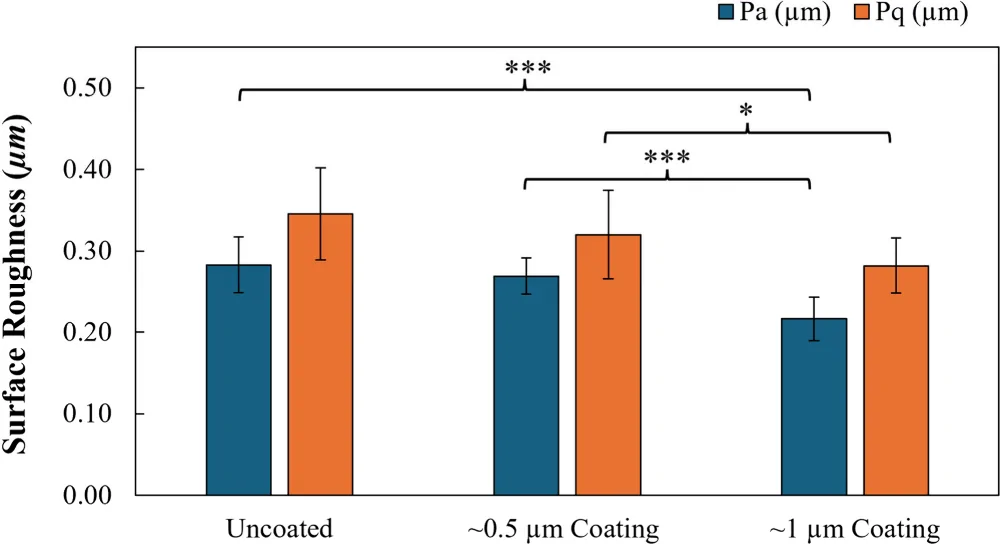
Surface roughness measurements of uncoated, ∼0.5 μm CaP and ∼1 μm CaP WE22 coupons (n = 3). * = p-value <0.05, *** = p-value <0.0001.

The optical micrograph preimmersions, Figure 5, illustrate the pearlescent nature of CaP coatings compared to the metallic surface of the uncoated Mg coupon. The visible changes in pearlescent color of coating further validate effective deposition and correlate to increased coating time and thickness.

**Figure 5:**
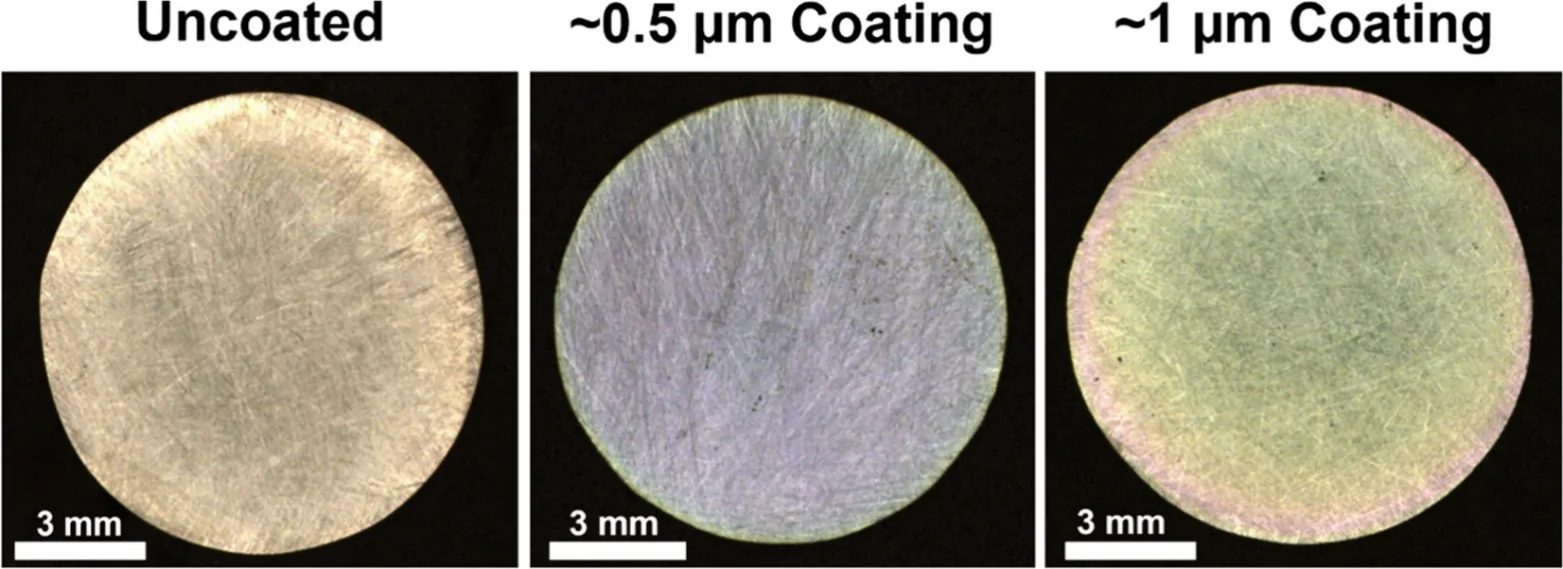
Optical microscopic images of uncoated, ∼0.5 μm and ∼1 μm CaP WE22 coupons pre immersion.

SEM micrographs of uncoated, ∼0.5 μm and ∼1 μm CaP coated WE22, Figure 6, show observable striations created on the alloy surface from the polishing process. It is of note that these striations start to in-fill and are less distinct not only when a coating is deposited but as the coating time, and subsequent thickness of coating, increases.

**Figure 6:**
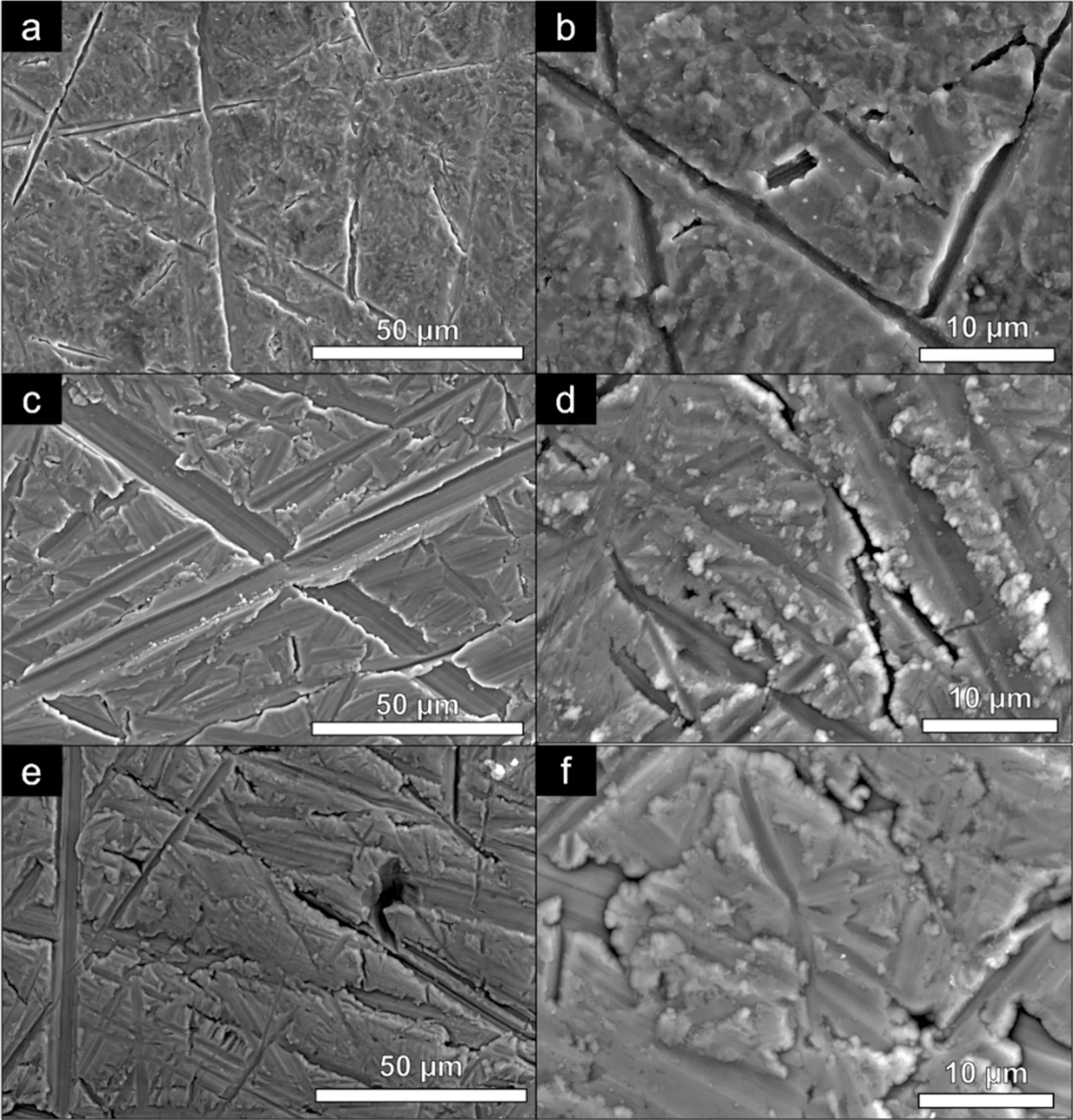
Micrographs of (a,b) uncoated WE22, (c,d) ∼0.5 μm CaP coated WE22, and (e,f) ∼1 μm CaP coated WE22.

EDX analysis presented quantitatively in Table 3 and qualitatively in Figure 7 shows the presence and elemental composition of Mg, Ca, and P in the uncoated and sputtered coating samples. It is evident that the Mg content in the samples significantly decreases, from ∼22% to ∼1%, as coating thickness increases. Conversely, the presence of Ca and P increases with thickness, from ∼19% to ∼26% and from ∼10% to 14%, respectively.

**Table 3:** EDX Elemental Data as Average Atomic % for Uncoated, ∼0.5 μm and ∼1 μm CaP Coated WE22 (n = 3)

**Element**	**Uncoated alloy (at.%)**	**∼0.5 Coating (at.%)**	**∼1 um Coating (at.%)**
Mg	85.1 ± 1.31	22.0 ± 0.23	1.1 ± 0.06
Y	0.63 ± 0.06	/	/
Ca	/	18.6 ± 0.57	25.6 ± 0.20
P	/	9.8 ± 0.58	13.7 ± 0.10
Ca/P Ratio	/	∼1.90	–1.87

**Figure 7:**
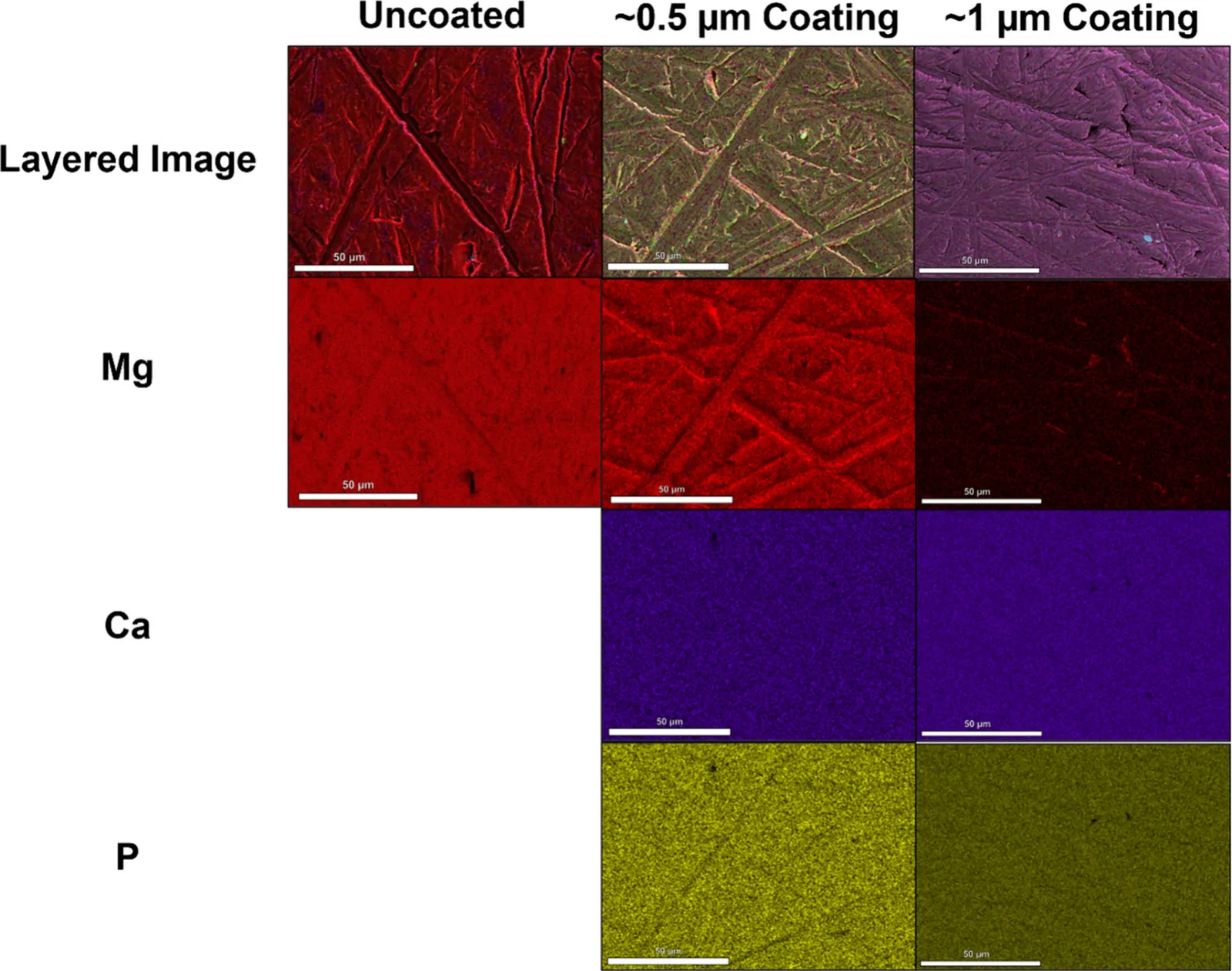
EDX qualitative maps displaying presence of Mg, Ca and P in uncoated, ∼0.5 μm and ∼1 μm CaP coated WE22.

Figure 8 demonstrates the mean mass gain for each of the sample types due to corrosion product formation after immersing in medium for 1, 7, and 24 h. After 1 h of immersion, ∼0.5 μm CaP coated samples exhibited significantly higher mass gain than both uncoated samples (*p* < 0.01) and ∼1 μm CaP coated samples (*p* < 0.05), indicating early corrosion product formation on the thinner coating. At 7 h, no statistical differences were observed between sample types; interestingly, the ∼1 μm CaP coated samples gained less mass (*p* < 0.01) post 1 h immersion. For uncoated and ∼1 μm CaP coated samples, there were no significant differences between the 7 and 24 h immersion time points. In comparison, ∼0.5μm CaP coated samples showed a substantial increase in mass gain at 24 h, with considerable variability between sample replicates.

**Figure 8:**
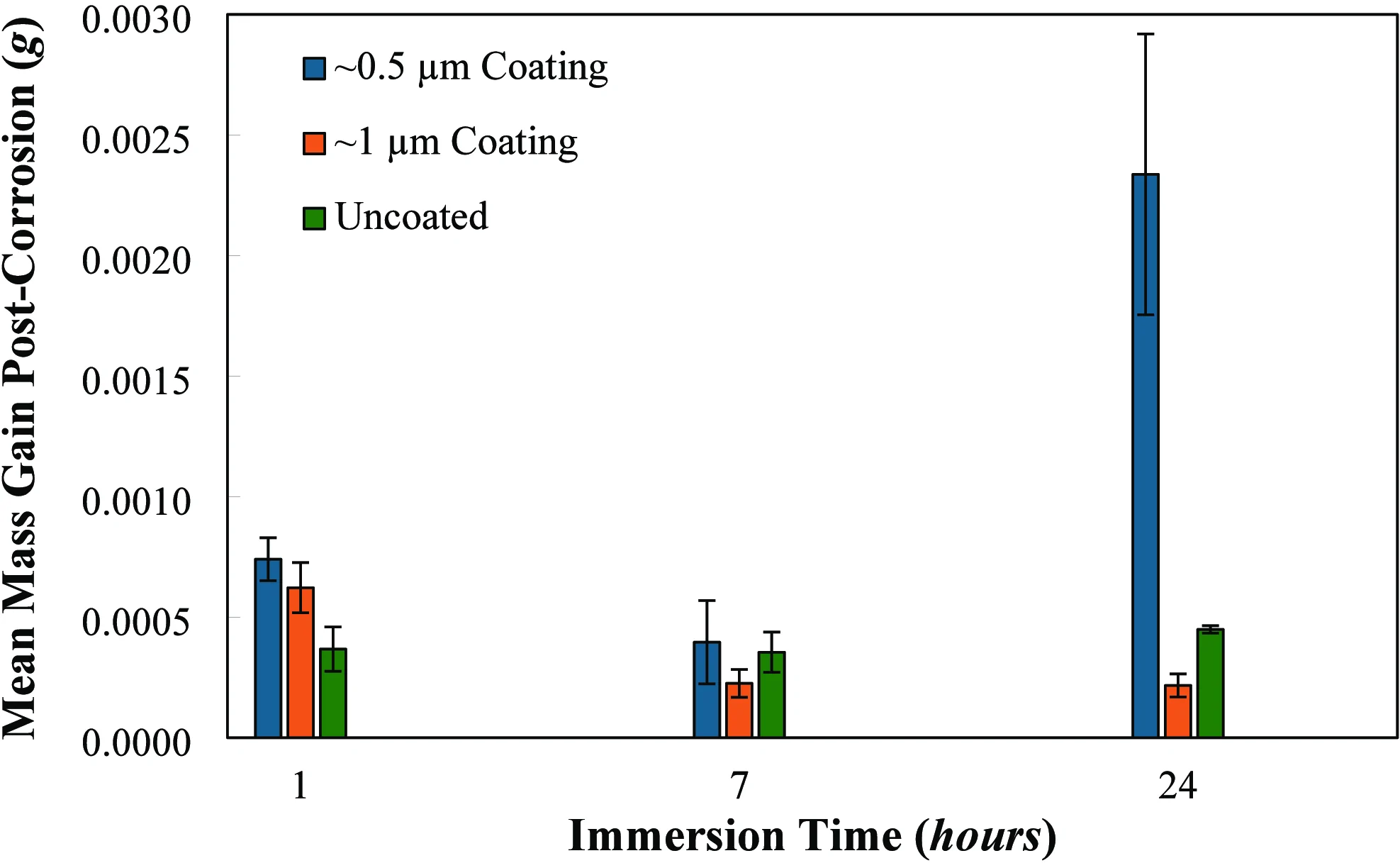
Mean mass gain of uncoated WE22, ∼0.5 μm and ∼1 μm CaP coated WE22 post-immersion (n = 3).

Representative optical micrographs of uncoated WE22 and CaP coatings on WE22 after exposure to McCoys 5A medium for 1, 7, and 24 h are exhibited in Figure 9. At all the investigated time points, the uncoated alloys have darkened regions and are indicative of localized corrosion, and this becomes more pronounced as the time in medium increases. In contrast, both the ∼0.5 μm and ∼1 μm CaP coated samples result in notably reduced surface corrosion, with the ∼1 μm coating exhibiting the most corrosion resistance over 24 h. This highlights the protective effect of CaP coatings against corrosion.

**Figure 9:**
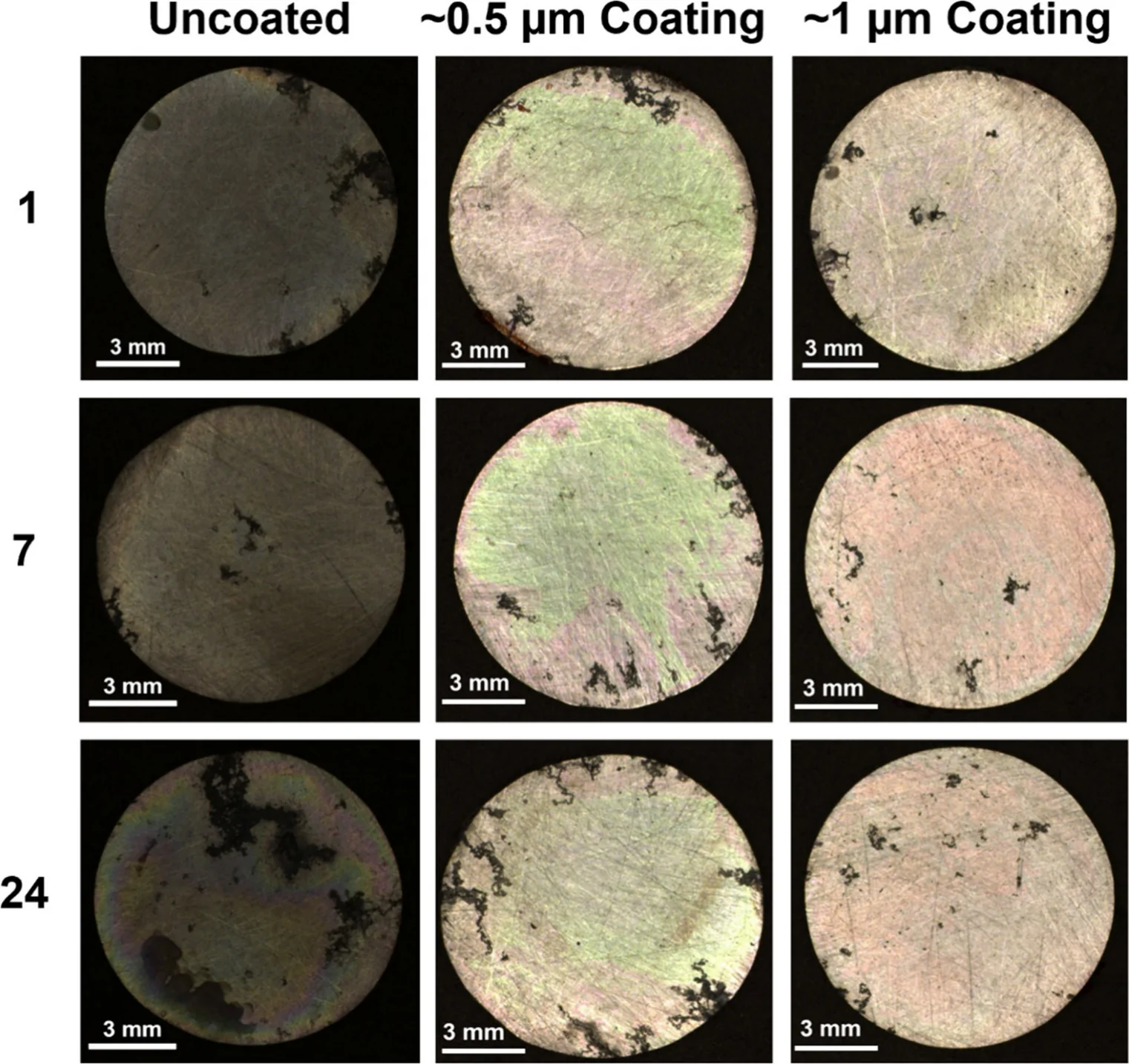
Optical microscopic images of uncoated, ∼0.5 μm and ∼1 μm CaP WE22 coupons post immersion in McCoys 5A medium for 1, 7, and 24 h.

The pH of McCoy’s 5A medium was measured prior to experimentation to determine any changes due to the corrosion process. The most notable differences in pH occurred after 24 h in medium, displayed in Table 4. Most evidently, the pH of medium collected from the uncoated WE22 samples increased to 8.8 ± 0.1, producing an environment with increased alkalinity. Although medium from samples of ∼0.5 μm exhibited an alkaline environment post-immersion, 8.1 ± 0.1, they reduced the shift towards higher alkalinity compared to uncoated WE22. Medium collected from ∼1 μm samples resulted in the least change in pH, 7.7 ± 0.2 after 24 h immersed, and approached close to a neutral and physiologically comparable environment.

**Table 4:** pH Measurements of McCoys 5A Medium Pre Immersion and Post 24 h Immersion with Uncoated, ∼0.5 μm and ∼1 μm CaP WE22 Coupons (n = 3)

**Sample Type**	**pH of medium (pre immersion)**	**pH of medium (post 24 h immersion)**
Uncoated WE22	7.1 ± 0.3	8.8 ± 0.1
∼0.5 μm Coating	″	8.1 ± 0.1
∼1 μm Coating	″	7.7 ± 0.2

FTIR-ATR analysis was conducted on the ∼0.5 μm and ∼1 μm CaP coatings post-immersion to assess corrosion products and adsorbed species on the substrate surface, Figure 10. The Amide I, II, and III bands are commonly attributed to protein vibrations, including C=O stretching (Amide I ∼1600-1700 cm^–1^)^22^ and N–H bending/C–N stretching (Amide II ∼1500-1600 cm^–1^ and Amide III ∼1300-1400 cm^–1^).^23,24^ The ∼0.5 μm CaP samples show a noticeably higher absorbance in the Amide region compared to the ∼1 μm CaP, suggesting greater protein adsorption or organic material on the surface. The phosphate region represents vibrations of PO_4_^3–^ that are attributed to amorphous CaP phases. The ∼1 μm CaP samples displayed a greater intensity at this region, which is expected from a thicker and more homogeneous coating.

**Figure 10:**
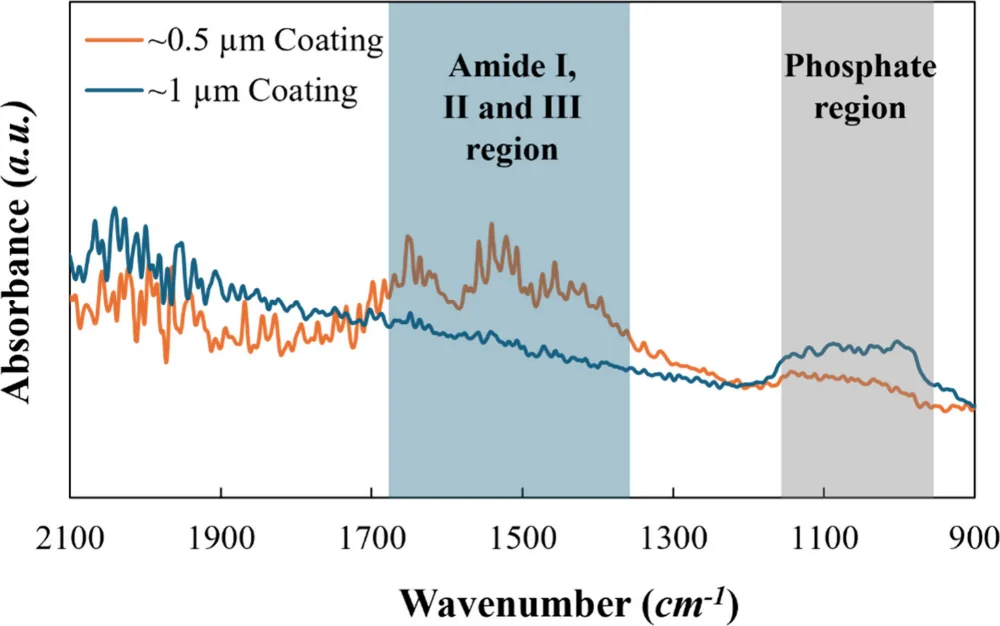
Representative FTIR-ATR spectra for ∼0.5 μm and ∼1 μm CaP coated WE22 alloys.

The concentration of Mg ions released from uncoated, ∼0.5 μm and ∼1 μm CaP coated WE22 samples is shown in Figure 11. At each timepoint and medium concentration, uncoated WE22 samples exhibited the highest Mg release, reaching a maximum concentration of ∼18 mg/L after 24 h in 100% corrosion medium. In contrast, both CaP-coated sample types showed notably reduced Mg release, with the ∼1 μm coating proving most effective in reducing ion release over 24 h.

**Figure 11:**
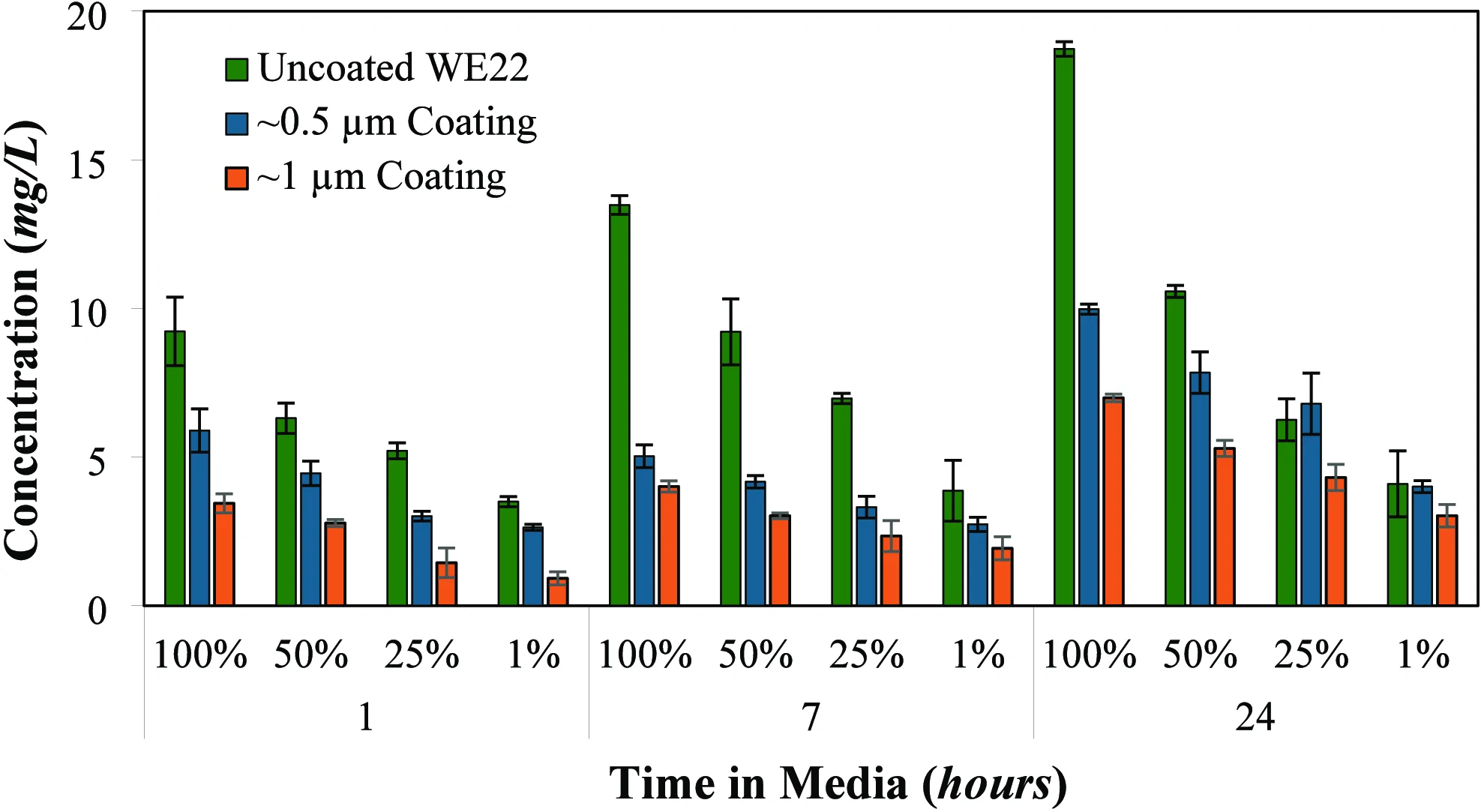
Mg concentration in corrosion medium at 1, 7, and 24 h.

Figure 12 illustrates the raw data of relative cell viability (%) assessed at multiple corrosion times (1, 7, and 24 h) using the corrosion medium from uncoated WE22, ∼0.5 μm and ∼1 μm CaP coated WE22 samples at various concentrations (100, 50, 25, and 1%). All samples were compared to positive controls, representing the optimal environment; any increase in relative cell viability (%) indicates a more positive impact on the cells. In all conditions, uncoated WE22 induced the greatest reduction in cell viability, with most evident effects at higher concentrations and longer corrosion times. Both CaP-coated samples exhibited significantly higher cell viability compared to uncoated WE22.

**Figure 12:**
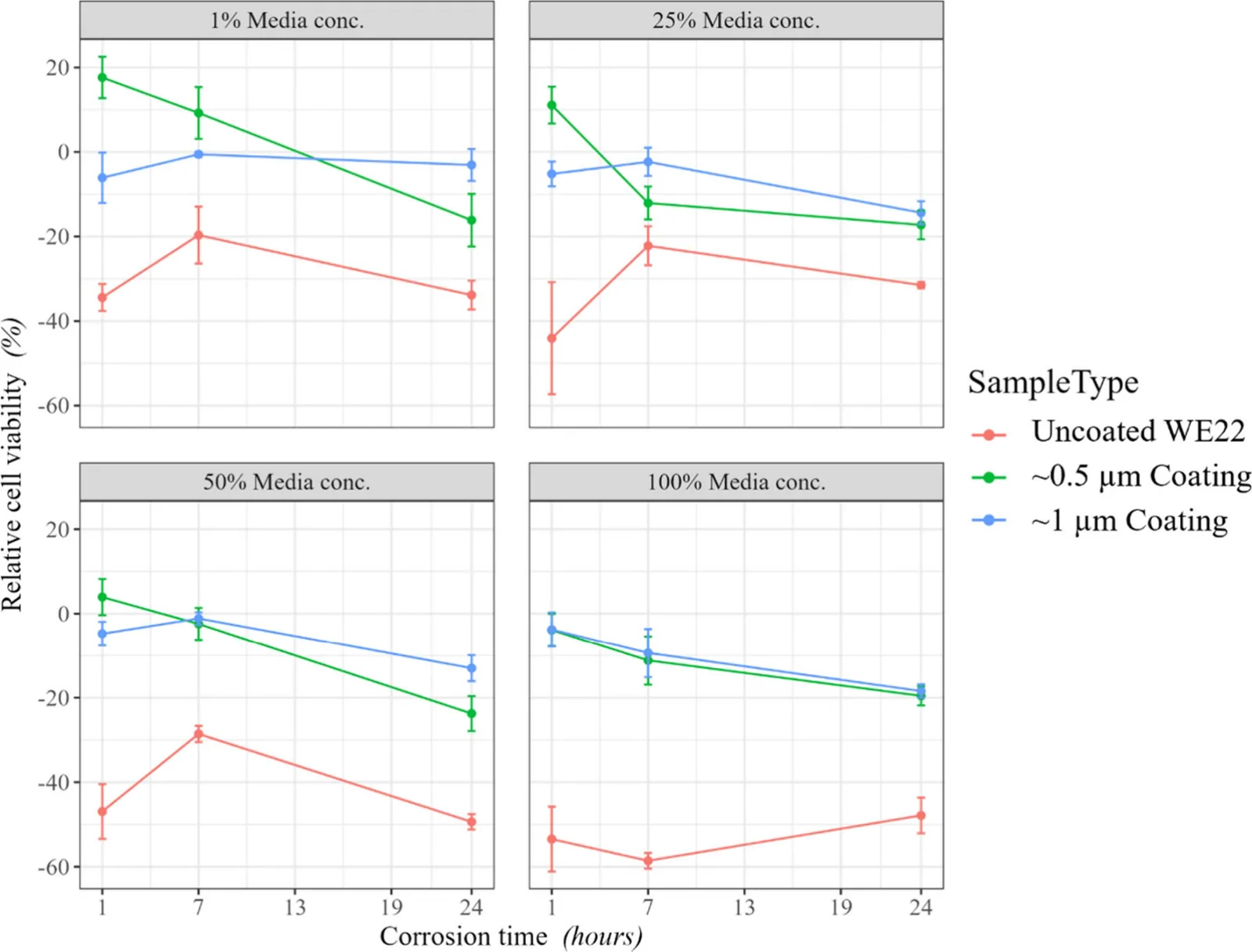
Raw data of Resazurin Reduction Assay results, indicating U-2 OS cell viability upon exposure to corrosion medium collected from uncoated, ∼0.5 μm CaP WE22 and ∼1 μm CaP WE22 samples at various concentrations (1, 25, 50, and 100%), cultured under standard conditions for 1, 3, and 7 days (n = 3).

The linear regression, presented in Figure 13, was used to model relative cell viability (%) as a function of corrosion medium concentration, sample type, and corrosion time. The R^2^ for the model was 0.78 showing that 78% of the variance in relative viability was explained by the sample type, corrosion time, and medium concentration. Cell viability decreased with increasing corrosion medium concentration independent of sample type or corrosion time, with each 10% increase in concentration resulting in ∼1.5% reduction in viability (*p* < 0.001). At 1 h corrosion time (and averaged over medium concentration), uncoated WE22 samples demonstrated substantially lower cell viability relative to the positive control (−39.5%, p < 0.001 for difference from zero), and viability for these samples did not meaningfully change with increasing corrosion time (slope not significant). The ∼0.5 μm CaP coating demonstrated the highest viability, with a predicted relative viability of +11.7% at 1 h corrosion in 1% medium (p < 0.001 vs reference). Viability for this coating decreased with corrosion time, at a rate of −1.1% per hour (p < 0.001). The ∼1 μm CaP coating showed similar behavior to the ∼0.5 μm coating, with a lower baseline viability at 1 h corrosion in 1% medium (+3.2%, p = 0.2 vs reference), and a slower reduction with corrosion time (slope −0.4% per hour, p = 0.03). Overall, the model confirms that the coating improves initial biocompatibility compared with uncoated WE22, but longer corrosion exposure reduces this benefit. This effect is most pronounced for the ∼0.5 μm CaP coating, which exceeds the positive control at low corrosion times.

**Figure 13:**
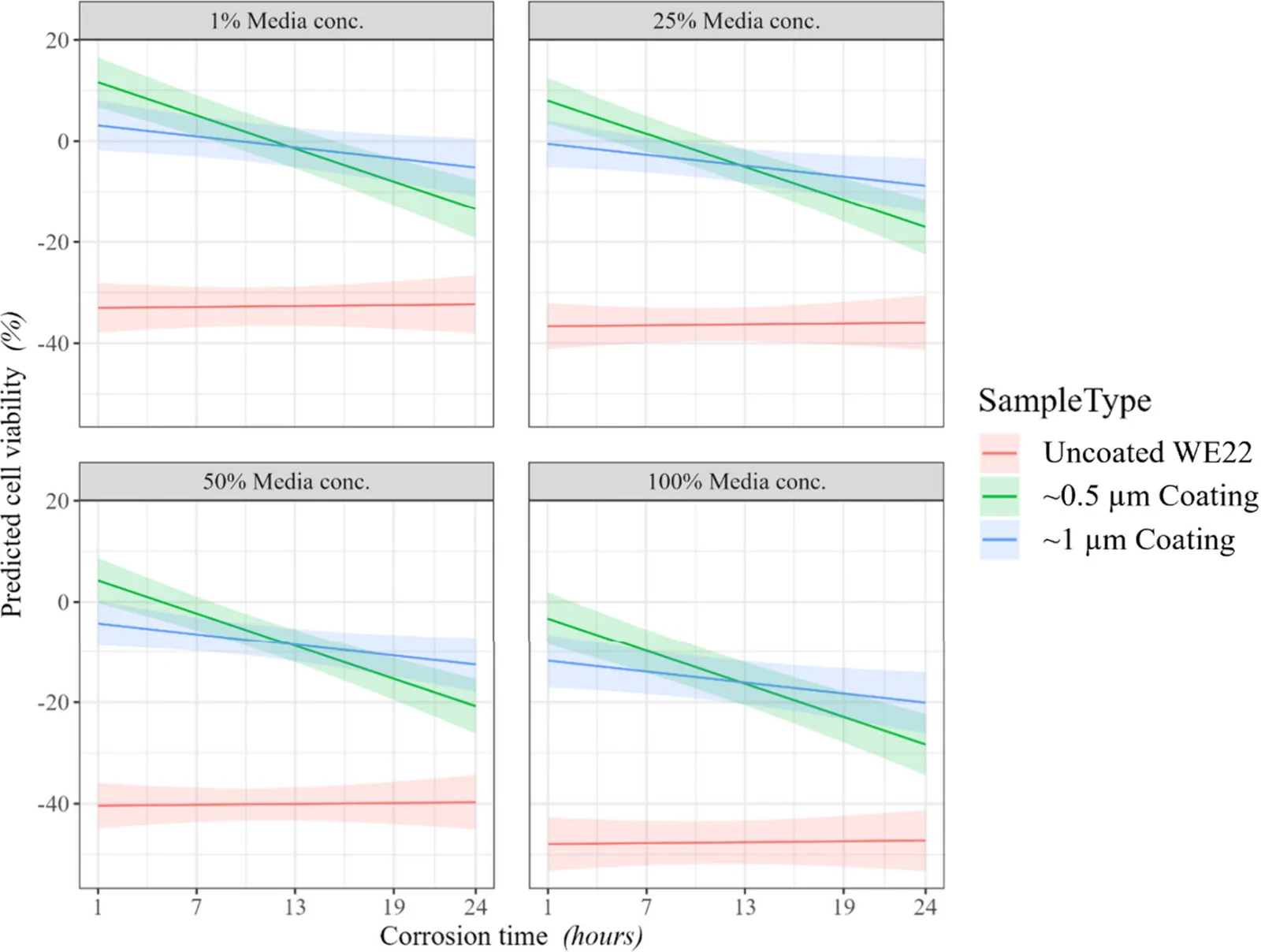
Predictions from linear regression model, R^2^ = 77%.

DAPI analysis, Figure 14, shows the fluorescent micrographs of DAPI-stained nuclei at day 7 in culture for uncoated, ∼0.5 μm and ∼1 μm CaP coated samples at the various media concentrations. Across all media concentrations, the uncoated WE22 consistently exhibits lower cell density and coverage compared to both CaP coated sample types. In contrast, both ∼0.5 μm and ∼1 μm CaP samples displayed higher and more uniform cell density, with sustained coverage (Table 5), even at higher media concentrations.

**Figure 14:**
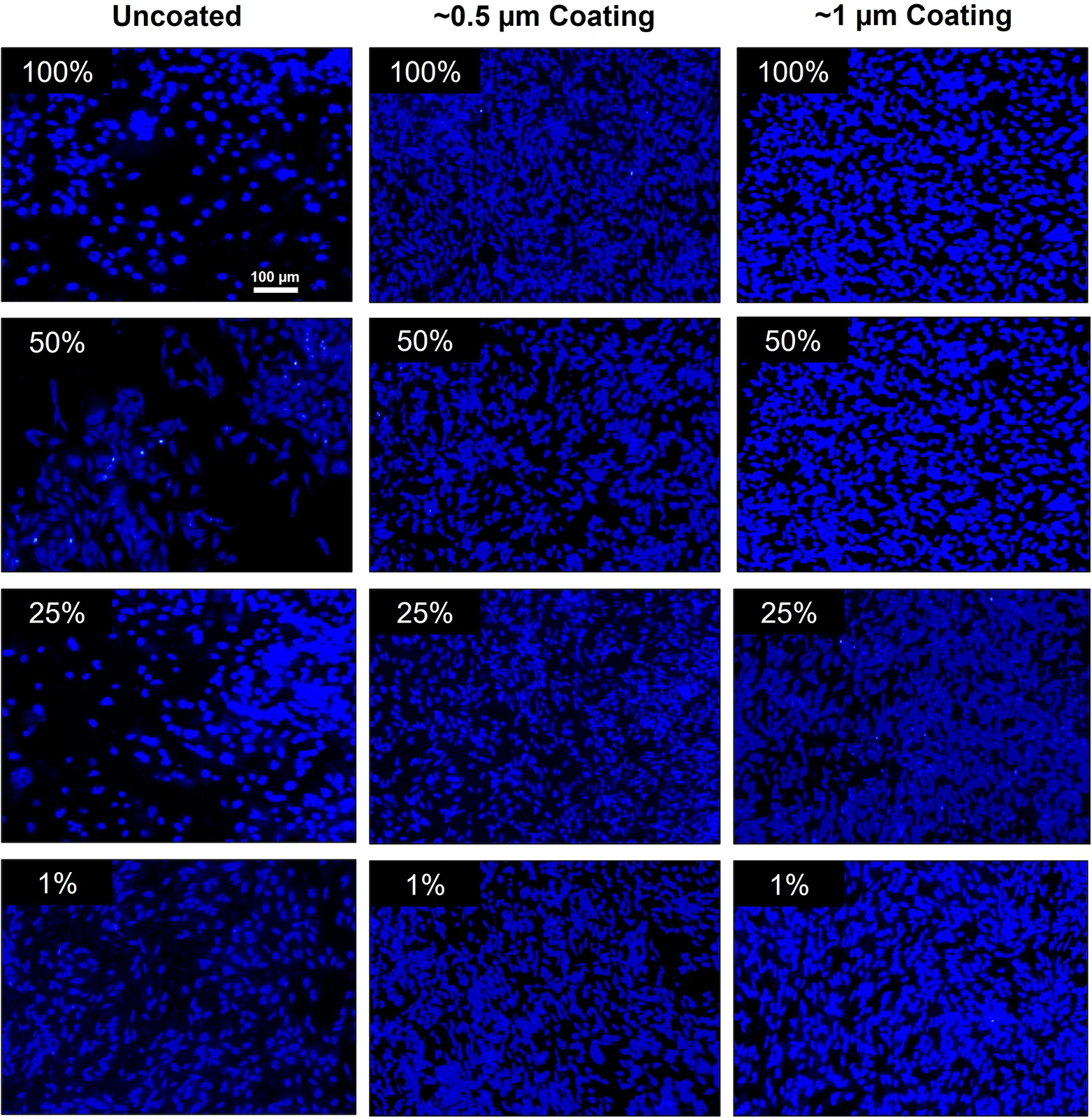
DAPI staining of U-2 OS cells cultured for 7 days with corrosion medium from uncoated WE22, ∼0.5 μm CaP WE22 and ∼1 μm CaP WE22 at varying concentrations (1, 25, 50, and 100%).

**Table 5:** Calculated % Coverage of U-2 OS Cell Monolayer from DAPI Stained Substrates

**Sample Type**	**Medium Concentration**	**% Coverage**
Uncoated WE22	100	18.99
	50	19.76
	25	23.67
	1	30.12
∼0.5 μm Coating	100	40.07
	50	36.10
	25	39.67
	1	45.17
∼1 μm Coating	100	46.71
	50	44.29
	25	48.10
	1	49.07

Adhered U-2 OS cells on each sample type were examined via SEM at day 7, Figure 15, to provide qualitative information on morphology. Cells fed with 100% corrosion media from CaP coated samples (∼0.5 μm and ∼1 μm thickness) exhibited a pronounced flattening morphology and have formed a dense film across the surface of the glass coverslip. Although there are cell adhesion and film-like properties on glass coverslips fed with uncoated corrosion medium, there are more apparent rounded and spherical cells visible across the surface and would suggest cell shrinkage – a key characteristic of apoptosis where cells decease in volume, change shape, and break down.

**Figure 15:**
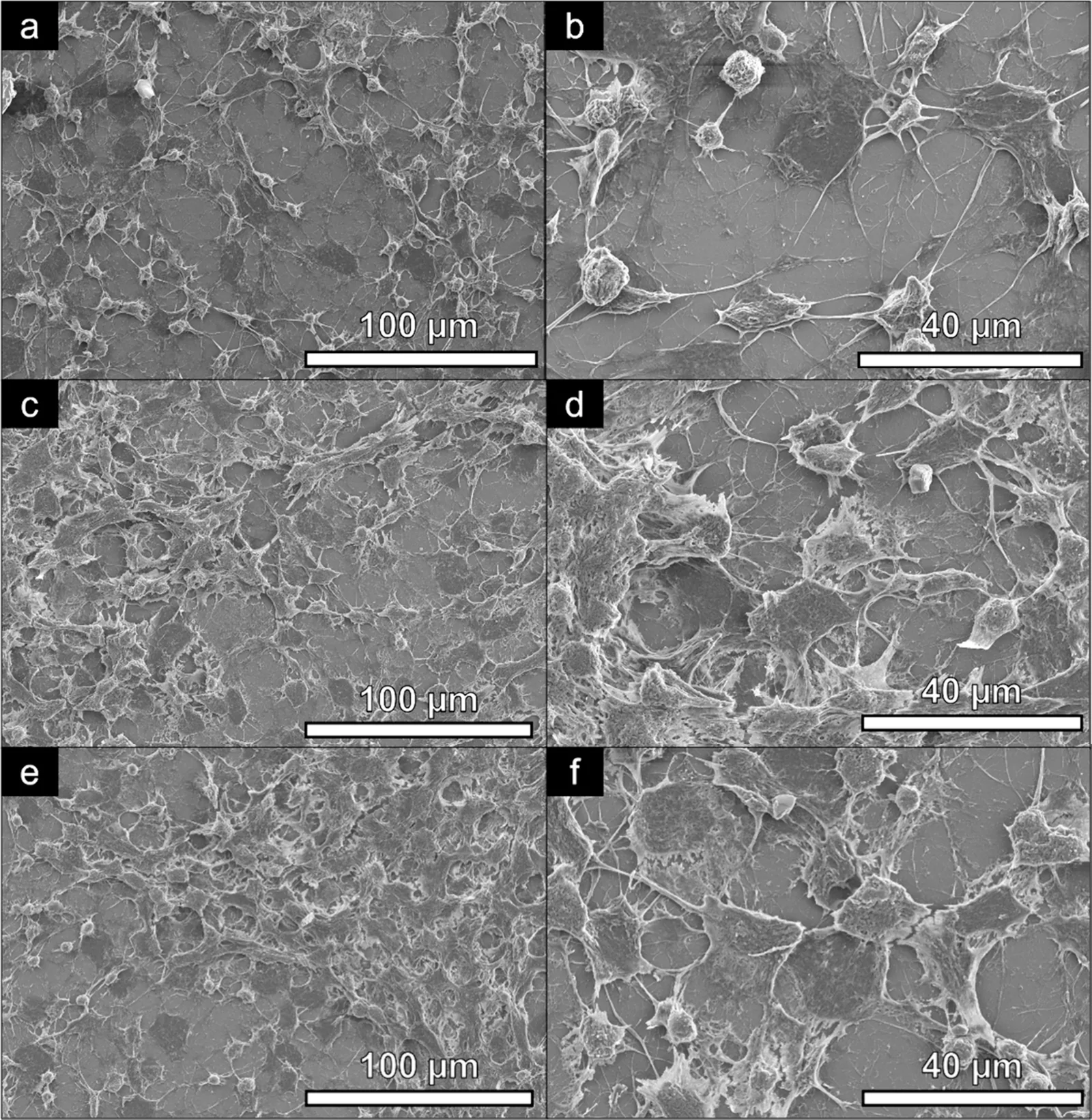
Micrographs of U-2 OS cells cultured on glass coverslips with 24-hour 100% dissolution medium derived from (a, b) uncoated WE22 alloy at x500 and x1.2k magnifications, (c, d) ∼0.5 μm CaP coated WE22 at x500 and x1.2k magnifications, and (e, f) ∼1 μm CaP coated WE22 at x500 and x1.2k magnifications.

The layered image obtained by EDX analysis shows the combined elemental mapping of the film and provides an overview of the presence and distribution of carbon (C) and oxygen (O), Figure 16. The widespread distribution of C is consistent with the organic content of the cell layers and supports the presence of a well-adhered film of U-2 OS cells.

**Figure 16:**
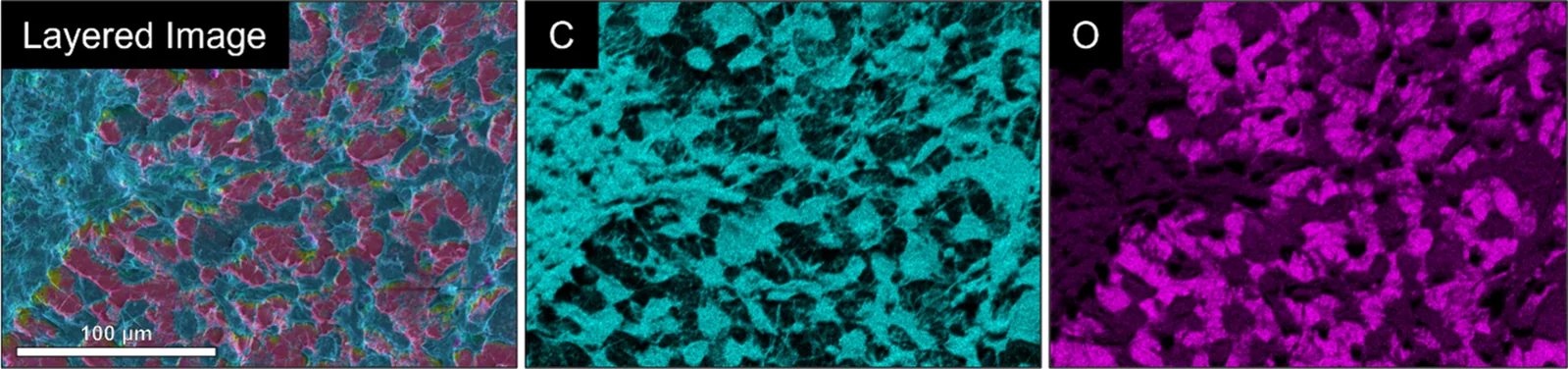
EDX analysis of U-2 OS film formed on the surface of glass coverslips cultured with corrosion medium from ∼0.5 μm CaP coating.

## 4 Discussion

### 4.1 Properties of CaP Coatings

As deposition time increased, the sputtered CaP coatings exhibited a linear growth pattern, Figure 1, which is consistent with previous findings employing RF Magnetron sputtering and other physical vapor deposition techniques under constant conditions^25^ – with linearity suggesting coating buildup is primarily linked to the deposition time. The formation of nanosphere-like features in sputtered amorphous CaP coatings, as shown in Figure 2, has also been previously documented in the literature^26^ and is largely influenced by the interplay between plasma energy, target composition, and various sputtering parameters. These thermodynamic and kinetic processes during deposition are thought to promote the sphere-like structures, as high-energy plasma species condense on the substrate and the lack of long-range crystalline order favors rounded structures that minimize surface free energy.^27,28^

GI-XRD results, Figure 3, further confirm that longer deposition times produced thicker coatings, as evidenced by the decreasing Mg peak intensity, as thickness increased. It also verifies the amorphous nature of the sputtered coating with the absence of HA-related crystalline peaks. In this respect, the present work not only aligns with well-established literature^26,29^ but also further demonstrates the consistent amorphous structure and same structural/morphological trends on a biodegradable WE22 alloy – a customized and medically relevant substrate less frequently examined. Notably, the spheroidal features of the amorphous coating become more pronounced and uniform at higher magnification and longer deposition, supporting the literature that links ‘snowballing’ features to film thickness.^30^

Longer deposition runs, of 30 h, produced ∼1 μm coatings with reduced surface roughness (Figure 4) compared to uncoated and 15-hour (∼0.5 μm thick) coated samples; this effect is attributed to the progressive infilling of surface striations and an overall smoother topography. In addition, the pearlescent changes evident across sample types (Figure 5) and the coating coverage illustrated in micrographs and EDX analysis (Figure 6 and Figure 7) further corroborate improved homogeneity and coverage with increased deposition time and thickness.

### 4.2 Degradation Behavior During Immersion

As shown in Figure 8, the ∼0.5 μm CaP coated samples exhibited the greatest mass gain after 24 h immersion, which may indicate accumulation of corrosion products and adsorption of organic deposits from the cell culture medium. Although Figure 9 illustrates the efficiency of CaP coatings improving corrosion resistance compared to the uncoated alloy, ∼0.5 μm coatings exhibited more prominent surface defects than the ∼1 μm coating, suggesting that the thinner coating was less effective as a barrier and may have undergone early-stage dissolution. In contrast, the ∼1 μm coatings maintained greater uniformity with fewer localized corrosion sites, indicating enhanced corrosion resistance. This observation may be explained by several contributing factors, with previous work on CaP coatings indicating that coating stability/dissolution strongly depends on thickness,^31,32^ substrate surface topography,^33,34^ and post-deposition treatment.^35^ Thinner CaP coatings are generally more susceptible to defects caused by non-uniform deposition or hydrogen gas evolution,^36^ both of which can promote nucleation sites for corrosion.^12^ Simultaneously, corrosion products may accumulate on the surface and could potentially exceed alloy dissolution and volume loss, resulting in an overall increase in mass. This phenomenon highlights the dynamic equilibrium when immersing substrates in physiologically relevant environments.

Additionally, organic constituents in fully supplemented McCoy’s 5A medium, including proteins and amino acids, are known to adsorb more readily onto coated surfaces,^37,38^ with this effect likely to be accelerated on a rougher or defected surface. Results from FTIR, Figure 10, further indicate adsorption of organics as ∼0.5 μm coated samples showed amide regions post-immersion, whereas these features were not evident on ∼1 μm samples. This suggests that the thinner coatings provided a more favorable surface for adsorbed organic components and entrapment of corrosion products if there were topographical defects present resulting in nucleation sites. Another topographical consideration for enhanced adherence on the surface of ∼0.5 μm samples is the previously discussed surface roughness. Protein adherence is generally enhanced on rougher surfaces, as the increased surface area offers more points for protein-surface interactions.^39,40^ The Pa and Pq roughness values for ∼0.5 μm samples were significantly more than the ∼1 μm samples (Pa = *p* < 0.01 and Pq = *p* < 0.05), suggesting increased protein contact and potential adhesion.

Corrosion of uncoated WE22, and Mg alloys in general, is a highly reactive and complex process resulting in various simultaneous reactions. Potential outcomes of corrosion are the formation of Mg hydroxide (Mg­(OH)_2_) and Mg oxide (MgO), as shown in the equations below:

$$Mg+2H_{2}O\to Mg{\left(\mathrm{OH}\right)}_{2}+H_{2}$$

$$Mg{\left(\mathrm{OH}\right)}_{2}\to MgO+H_{2}O$$

These corrosion products typically form a loosely bound film on the surface that is partially protective but may be readily dissolvable. As these corrosion products have limited stability in medium, uncoated WE22 samples are less likely to accumulate surface deposits to the same extent as the ∼0.5 μm samples.

### 4.3 Cell Viability

Indirect assessment of cell viability, utilizing medium collected from the degradation study, allowed assessment of toxic effects that may be caused by released ions, pH changes, and corrosion products without direct cell-to-metal contact. This approach is particularly relevant for biodegradable materials, such as WE22, due to the extreme physical and chemical changes occurring during the degradation process. When Mg is exposed to aqueous solutions, such as McCoys 5A medium, the corrosion mechanism of the metal itself is described as anodic, whereas the cathodic reaction involves the formation of hydroxide ions; these reactions are detailed below:

$$Mg\to {Mg}^{2+}+2e^{-}$$

$$2H_{2}O+2e^{-}\to H_{2}+2{\mathrm{OH}}^{-}$$

The formation of OH^–^ causes a rise in local pH at the alloy surface, increasing the alkalinity of the surrounding environment beyond relevant physiological conditions.^32^ Although storing solutions throughout this experimentation at 5% CO_2_, which aids in maintaining pH at physiological levels, the release of OH^–^ due to corrosion of the Mg alloy may exceed the buffering capacity of medium, leading to strong alkaline conditions and observable effects (storage in containers used may have limited CO_2_ exchange).

The pH measurements collected pre- and post-immersion, Table 4, of uncoated samples showed an increase in pH over 24 h from 7.1 ± 0.3 to 8.8 ± 0.1. Mg^2+^ release was reviewed upon removal of samples from medium, shown in Figure 11. The highest release was observed from uncoated WE22 samples, with a concentration of ∼18 mg/L released after 24 h of immersion. While increased Mg^2+^ may contribute to minor inhibitory effects on U-2 OS cells, the concurrent increase in pH caused by Mg corrosion can also play a substantial role.^41^ Elevated pH values above physiological levels have shown to induce alkaline stress, which can disrupt cellular processes and may trigger apoptosis.^42^ Both coated sample types released significantly less Mg^2+^ over 24 h in medium, with maximum concentrations measuring at ∼10 mg/L and 7 mg/L for ∼0.5 μm and ∼1 μm CaP coatings, respectively. Moreover, the efficiency offered by the ∼1 μm coating was further highlighted from the stability of medium pH where alkalinity was reduced and close to physiological conditions were maintained, Table 4. This was further highlighted in the biological assays conducted; the raw data from RR results (Figure 12) demonstrates that CaP-coated samples both significantly improved cell viability compared to uncoated WE22, confirming the protective effect of the coating in reducing corrosion, subsequent ion release, and cytotoxicity. A data point of note is the significant increase in cell viability observed for the ∼0.5 μm CaP coating after 1 h of corrosion and a medium concentration of 1% (+11.7% compared to positive control, *p* < 0.001). Overall, the ∼0.5 μm coatings displayed a range of variability in terms of localized dissolution and in mass gain/adherence to surface. At this specific timepoint and concentration of corrosion medium, the dissolute may have supported cellular metabolic activity and surpassed optimal conditions provided by the positive control. The linear regression model performed (Figure 13) predicted a ∼1.5% decrease (*p* < 0.001) in cell viability across all samples with a 10% increase in medium concentration. However, the rate of decrease was significantly reduced by the addition of both coating types due to the coatings acting as a barrier for corrosion. Qualitative and quantitative analyses performed from DAPI staining, Figure 14 and Table 5, corroborate these findings as they illustrate a decrease in cell density and coverage as concentration of corrosion medium increases – this is most extensive in uncoated samples. Cell attachment, flattening, and proliferating into a monolayer was evident with substrates cultured in medium from coated samples, Figure 15; evidence of a monolayer was confirmed with EDX analysis in Figure 16. The round and spherical morphology visible in cells cultured in uncoated corrosion medium indicates potential cytotoxicity. Overall, these findings emphasize that CaP coatings, including their thickness and uniformity, can maintain biocompatibility by limiting Mg corrosion and associated reactive factors.

## 5 Conclusion

The effect of initial dissolution behavior on cellular response and the control of WE22 corrosion, through the application of CaP coatings, has been investigated. RF magnetron sputtered HA targets were utilized to fabricate CaP coatings at two thicknesses, ∼0.5 μm and ∼1 μm, to evaluate against an uncoated WE22 alloy. Coating deposition times corresponded to approximate coating thicknesses, measured by line profilometry measurements, of ∼0.5 μm and ∼1 μm for 15 and 30-hour coatings, respectively. The amorphous nature of the CaP coatings was confirmed via XRD analysis with micrographs from SEM detailing the ‘snowball’ features associated with sputtering amorphous HA. The reactive nature of uncoated WE22 contributed to early-stage corrosion, significant Mg^2+^ release (∼18 mg/L after 24 h in medium), and an increase in alkalinity from physiologically relevant conditions (from ∼7 to ∼8.8). Interestingly, the ∼0.5 μm CaP coating did reduce these effects but did, however, produce the greatest mass gain post-immersion, with FTIR analysis indicating adhesion of organic components onto the surface of the substrate. This may be due to potential nano-defects on coating and inhomogeneities that may be trapping corrosion products and constituents from the medium. These same entrapments may not occur in uncoated WE22 due to the unstable nature of oxide/hydroxide layers that disrupt similar entrapment. Samples of ∼1 μm thickness were most stable and unreactive throughout immersion in medium, enhancing corrosion resistance, minimizing changes in alkalinity, and decreasing the magnitude of surrounding Mg^2+^ ions. RR assay, DAPI staining, and SEM assessment of U2-OS cells cultured with the varying corrosion media all indicated that cell viability decreased significantly with increasing medium concentration (*p* < 0.001); however, cells cultured with medium from uncoated WE22 were significantly more detrimental. The coverage (%) of nuclei, calculated from DAPI, aligned with these findings, and coverage from uncoated samples reduced most significantly from 1% corrosion medium to 100%, with a reduction of ∼11% compared to ∼5% and 2% for ∼0.5 μm and ∼1 μm CaP coated samples, respectively.

## Supplementary Material



## Notes

Further details regarding AAS calibration (PDF)
