# Supplementary material for: Effect of Initial Dissolution Behavior on Cellular Response in Uncoated and Calcium Phosphate-Coated Magnesium Alloy
Source: ACS Omega. 2026 Sep 30;11(40):60848–61. doi: 10.1021/acsomega.6c04124 (PMC13670756; doi:10.1021/acsomega.6c04124)
Supplement: Supplementary file 1 [file ao6c04124_si_001.pdf]

Effect of Initial Dissolution Behaviour on Cellular Response in Uncoated and Calcium Phosphate Coated Magnesium Alloy

Aoife McFerran[a*], Adam Griebel[b], Jeremy Schaffer[b], Ellamay McIlhatton[a], John O'Connor[a], Joanna Ward[a], Jonathan G. Acheson[a]

[a] School of Engineering, Ulster University, Belfast, BT15 1AP, United Kingdom

[b] Fort Wayne Metals, Fort Wayne, Indiana, 46809, United States

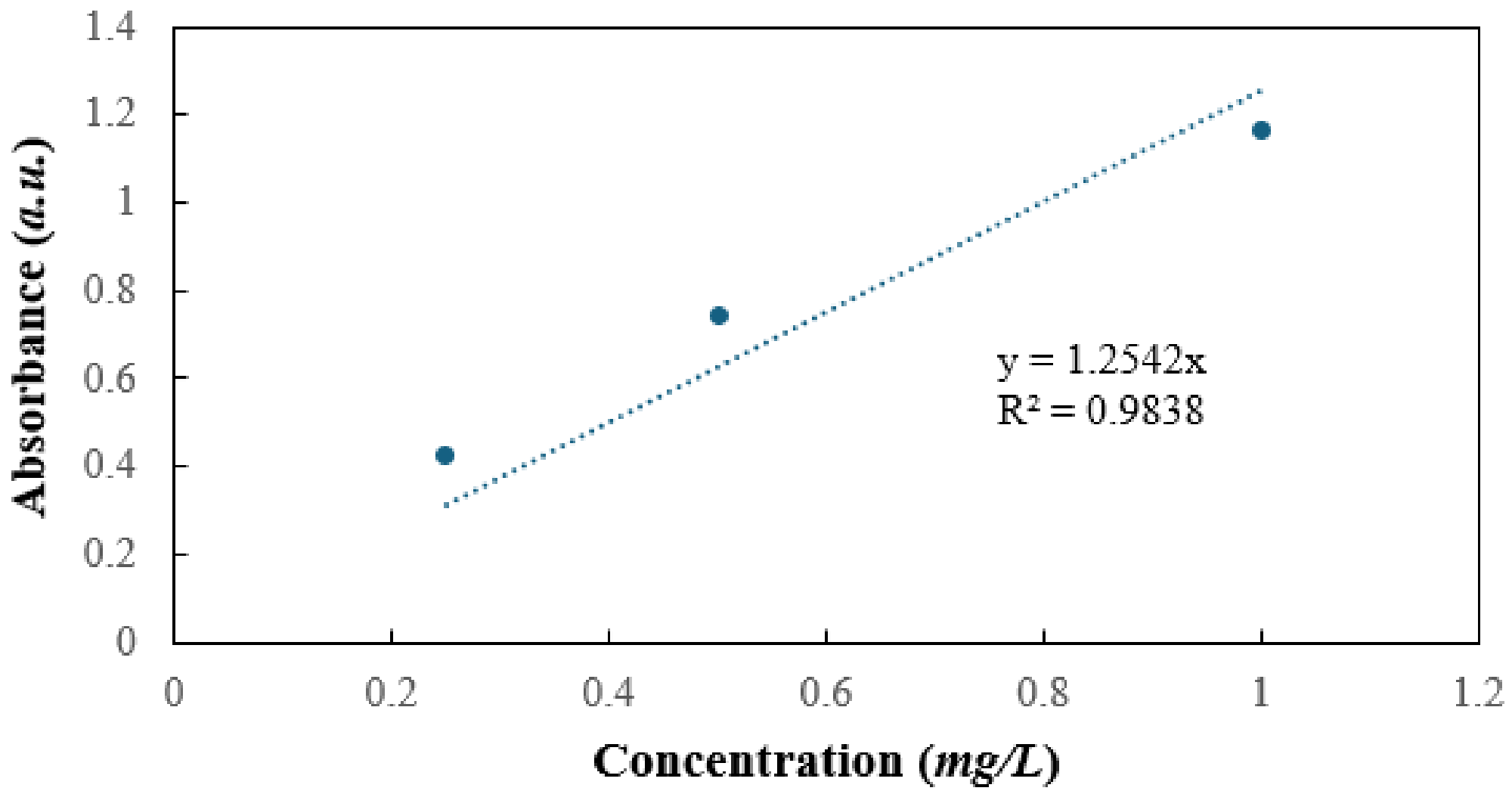

Figure 1 *Calibration curve of Mg stock solutions for AAS analysis.*

```
Call: lm(formula = RelVia ~ CorrTime * SampleType + MedConc, data = data)

Residuals:
    Min      1Q  Median      3Q     Max
-33.948  -6.670   1.439   5.456  22.586

Coefficients:
                                  Estimate Std. Error t value Pr(>|t|)
(Intercept)                      -32.89573    2.60130 -12.646  < 2e-16 ***
CorrTime                           0.03019    0.16316   0.185   0.8536
SampleType15hr CaP WE22           45.78631    3.33321  13.736  < 2e-16 ***
SampleType30hr CaP WE22           36.53177    3.33321  10.960  < 2e-16 ***
MedConc                           -0.15080    0.02502  -6.028 2.73e-08 ***
CorrTime:SampleType15hr CaP WE22  -1.11992    0.23075  -4.853 4.43e-06 ***
CorrTime:SampleType30hr CaP WE22  -0.39101    0.23075  -1.695   0.0932 .
---
Signif. codes:  0 ‘***’ 0.001 ‘**’ 0.01 ‘*’ 0.05 ‘.’ 0.1 ‘ ’ 1

Residual standard error: 9.536 on 101 degrees of freedom
Multiple R-squared:  0.788,	Adjusted R-squared:  0.7754
F-statistic: 62.58 on 6 and 101 DF,  p-value: < 2.2e-16
```

Figure 2 *Full results of regression model.*
